# Ductal Carcinoma *In Situ*: What the Pathologist Needs to Know and Why

**DOI:** 10.1155/2013/914053

**Published:** 2013-02-06

**Authors:** Anita Bane

**Affiliations:** ^1^Department of Pathology and Molecular Medicine, McMaster University, Hamilton, ON, Canada L8V 1C3; ^2^Department of Oncology, McMaster University, Hamilton, ON, Canada L8V 1C3

## Abstract

Ductal carcinoma *in situ* is a proliferation of malignant epithelial cells confined to the ductolobular system of the breast. It is considered a pre-cursor lesion for invasive breast cancer and when identified patients are treated with some combination of surgery, +/− radiation therapy, and +/adjuvant tamoxifen. However, no good biomarkers exist that can predict with accuracy those cases of DCIS destined to progress to invasive disease or once treated those patients that are likely to suffer a recurrence; thus, in the era of screening mammography it seems likely that many patients with DCIS are overtreated. This paper details the parameters that should be included in a pathology report for a case of DClS with some explanations as to their importance for good clinical decision making.

## 1. Definition

Ductal carcinoma *in situ* (DCIS) is defined as a proliferation of malignant epithelial cells that has not breached the myoepithelial layer of the ductolobular system. DCIS is a highly heterogeneous disease in terms of presentation, morphology, biomarker expression, underlying genetic alterations, and natural progression. It is considered a precursor lesion with a relative risk (RR) of 8–11 for the subsequent development of invasive carcinoma [1]. In most cases DCIS involves the breast in a unicentric segmental fashion and true multicentric disease is unusual occurring in an estimated 10% of cases. 

## 2. Epidemiology

DCIS currently comprises ~20–25% of all newly diagnosed breast cancers in North America up from ~5% of cases in the early 1980's [2, 3]. This large increase in incidence is largely ascribed to the introduction of screening mammography. Currently 80–85% of DCIS cases are detected by mammography and the remainder are detected as a palpable lump or nipple alteration/discharge [2]. Interestingly the incidence of DCIS in women >50 years of age has been in decline since 2003, a fact that may be related to the declining use of postmenopausal hormonal therapy, whereas the incidence of DCIS continues to rise for women less than age 50 [4]. In addition to the dramatic rise in the incidence of DCIS detection, the introduction of screening mammography has led to a decline in mortality rates from DCIS; the death rate from DCIS diagnosed between 1978 and 1983 (prescreening mammography) was 3.4% at 10 years as compared to 1.9% at 10 years with DCIS diagnosed between 1984 and 1989 (screening era). Additionally, the spectrum of DCIS diagnosed has changed with the use of screening with more low and intermediate grade DCIS being diagnosed while the relative proportion of high grade DCIS has decreased [5]. 

Risk factors for the development of DCIS are similar to those for invasive breast cancer suggesting that both diseases are etiologically related and include increasing age (mean age at diagnosis for DCIS is 50–59 years), family history of a first degree relative with breast cancer, nulliparity or late age of first birth, late age of menopause, long-term use of postmenopausal hormonal therapy, elevated body mass index (BMI) in postmenopausal women, BRCA mutational status, and high mammographic breast density [6, 7]. The relationship of ethnicity to DCIS is currently under intense investigation.

## 3. Imaging and DCIS

DCIS commonly presents mammographically with calcifications either of the laminated (usually associated with lower grade DCIS) or amorphous/pleomorphic variety (more commonly associated with high-grade DCIS). A minority of mammographically detected DCISs (<20%) are associated with masses or areas of architectural distortion and mammography has been shown to commonly underestimate the extent of DCIS by up to 1-2 cm compared with definitive histology. MRI can detect high-grade DCIS but is unreliable for the detection of lower grade lesions.

## 4. Gross Pathology

Typically DCIS does not produce a lesion clearly identifiable on macroscopic examination of the resected specimen; exceptions include cases of high-grade DCIS with comedo necrosis in which expanded duct spaces with “soft centres” may be visible and some cases of solid papillary DCIS which may result in a mass lesion of variable size with a circumscribed margin.

## 5. Classification of DCIS

Despite the prevalence of DCIS there is no uniformly accepted classification system, however, a growing consensus of opinion recognises the importance of grade over morphology [8]. 

### 5.1. Grading of DCIS

 There are three commonly referenced grading schemes for DCIS, all of which employ the assessment of nuclear grade and presence/type of necrosis with some additionally utilising cellular polarity to ascribe an overall grade [9–11]. No one system has been endorsed; however, a consensus conference and the College of American Pathologists recommend that a pathology report should include a description of *nuclear* grade, presence and type of necrosis, and the architectural patterns present [8, 12]. Thus when we discuss the “grade” of DCIS this is now generally accepted to refer to the *nuclear* grade of the lesion. 

 Three nuclear grades are identifiable low (1), intermediate (2), and high (3). 

*Grade 1 (Low Grade)*

**Figure 1:**
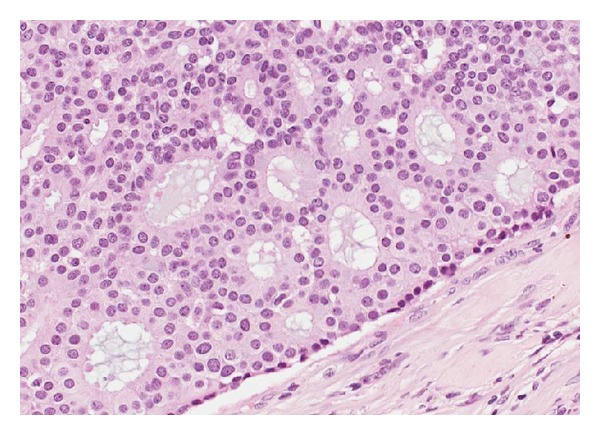
Low-grade DCIS. The neoplastic cells show small uniform nuclei with fine chromatin and are polarized around secondary lumina.

The nuclei are monomorphous and 1.5 to 2 times the diameter of a RBC with inconspicuous nucleoli and diffuse chromatin. The nuclei are usually orientated (polarized) toward the lumen (Figure 1).

*Grade 2 (Intermediate Grade)*

The nuclei are neither 1 nor 3.

*Grade 3 (High Grade)*

**Figure 2:**
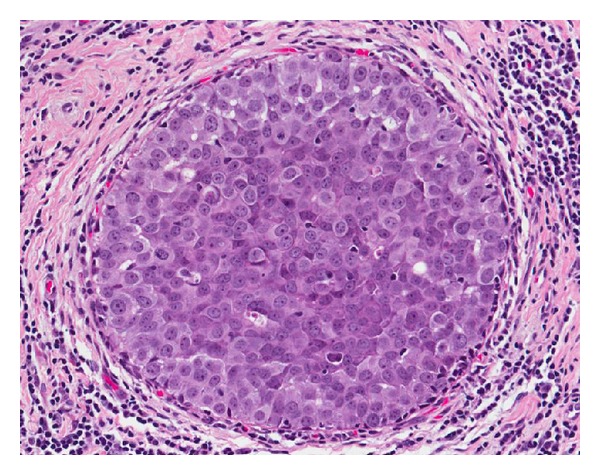
High-grade DCIS. The neoplastic cells demonstrate markedly enlarged nuclei, with significant pleomorphism, coarse chromatin, and lack of polarity.

The nuclei are large and pleomorphic, >2.5 times the diameter of a RBC with more than one nucleolus per cell, and contain irregular chromatin. The nuclear orientation is usually irregular (nonpolarized) (Figure 2).

 Two types of necrosis are identifiable.

*Comedo Type*

**Figure 3:**
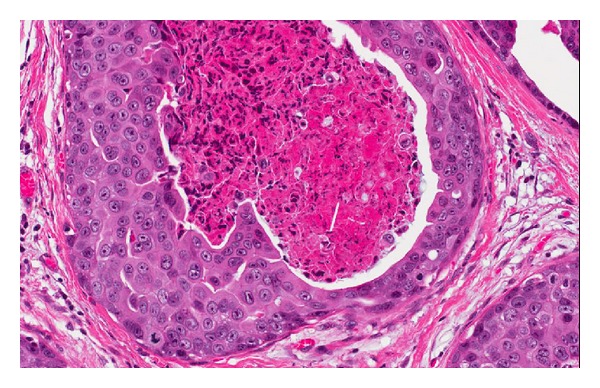
High-grade DCIS with central comedo-type necrosis.

Central areas of necrosis, ghost outlines of cells and cellular debris (Figure 3). 

*Non-Comedo Type*

Individual cell necrosis usually in the form of apoptotic cells.

In reality most substantive cases of DCIS show a variety of grades within the same lesion. Allred and colleagues have shown in a series of 120 cases of pure DCIS that 45.8% of cases showed areas of diversity with regard to nuclear grade (NG); 30% of cases contained areas of NG 1 and 2, 6.6% had an admixture of NG 2 and 3, and 9.2% had a mixture of NG 1, 2, and 3 [13]. While there are no clear guidelines on how to manage this scenario, in general one should grade to the “highest” grade within the lesion, if it composes a significant component (>10%) of the case. Interobserver agreement in assigning a grade is moderate at best.

 An alternate grading system for all atypical and malignant intraductal proliferations to include DCIS has been proposed by Tavassoli [14]. Using this system the term ductal intraepithelial neoplasia (DIN) replaces descriptive terms commonly used for atypical and malignant intraductal proliferations and a graduated numerical and alphabetical designation is employed to assign lesion severity; as such low-grade DCIS is termed DIN 1C, intermediated grade DCIS is termed DIN II, and high-grade DCIS is referred to as DIN III. While this system has many merits it has not been widely adopted to date in the clinical community. 

### 5.2. Morphological Variants of DCIS

 There are many morphological variants of DCIS including comedo, solid, clinging, cribriform, papillary, solid variant of papillary DCIS, micropapillary, neuroendocrine, apocrine, cystic secretory, and Pagets disease. A significant proportion of DCIS lesions will harbour more than one morphological variant and all variants should be mentioned in the final synoptic report. Most of these morphological variants are well known to practicing pathologists but some are sufficiently rare or have some caveats that deserve mention.

*Solid Papillary Carcinoma and Encysted (Intracystic) Papillary Carcinoma*

While these entities have traditionally been considered as variants of DCIS recent studies have shown that when immunohistochemical markers for myoepithelial cells are employed some lesions have been found to lack such cells around the periphery of the tumor; in such cases whether the lesion is truly DCIS or a low-grade invasive carcinoma with pushing tumor margins is unresolved. Rakha and colleagues have recommended considering those lesions with demonstrable myoepithelial cells as DCIS and those without as a special type of invasive carcinoma. In their study, these lesions were associated with a low incidence of stromal/skeletal muscle invasion, low frequency of lymph node metastasis (3%), and infrequent development of local or distant recurrence. Thus they conclude that these lesions are characterized by indolent behaviour and extremely favourable prognosis. They go on to stress that these lesions can be treated with adequate local therapy without the need for adjuvant chemotherapy [15].

*Micropapillary DCIS*

Micropapillary carcinoma is a variant of DCIS characterised by the presence of intraluminal tufts of malignant cells that lack a true fibrovascular core. When present in a “pure” form, that is, not admixed with other morphological variants, it has been shown in one study to be associated with extensive disease involving multiple quadrants [16].

*Apocrine DCIS (ADCIS)*

Scott et al. recommended that apocrine DCIS be recognized as a special variant of DCIS given its rarity, the specific challenges in distinguishing it from atypical apocrine proliferations, and the difficulties with assigning an accurate grade [11]. Many morphological variants of apocrine DCIS have been described including solid, cribriform, and comedo subtypes but it is the characteristic cellular features of large cells with abundant eosinophilic cytoplasm with enlarged nuclei and prominent nucleoli that define this entity. 

Nuclear grading of apocrine DCIS is particularly difficult as classic apocrine cells are enlarged with prominent nucleoli relative to normal breast epithelium leading some authors to suggest that the diagnosis and grading of apocrine DCIS specifically should rely not only on nuclear grade and the presence/type of necrosis but also on the size of the lesion such that low-grade apocrine DCIS is present when the apocrine cells are 3-4X the size of benign apocrine cells, necrosis is absent, and the size of the proliferation of concern is at least 4–8 mm. 

High-grade apocrine DCIS is present when the apocrine cells are ≥5 times the size of benign apocrine cells and comedo-type necrosis is present. And when these two criteria are satisfied a minimum size criterion is not necessary.

Intermediate-grade apocrine DCIS describes those lesions that have nuclei in the size range of low-grade ADCIS that is, 3-4X a benign apocrine cell but have comedo-type necrosis or those lesions with apocrine cells typical of high-grade disease (≥5 times the size of a normal apocrine cell) but comedo-type necrosis is *not *evident [17–19].

 Given the rarity of these lesions in clinical practice a consensus opinion or expert referral may be warranted.

*Cystic Hypersecretory DCIS*

This is an extremely rare variant of DCIS characterised by cystically dilated ducts line by a mixture of benign, hyperplasic and malignant epithelium with micropapillary, and cribriform-type arrangements. The cells may have vacuolated cytoplasm reminiscent of lactating epithelium and stain positively for mucin. The cyst lumens often contain a viscous colloid secretary-type material [20, 21].

*Paget's Disease*

Paget's disease of the nipple is characterized by the presence of malignant epithelial cells within the squamous epithelium of the nipple-areola complex. It often presents as an eczematous change in the nipple-areola area and is invariably associated with high-grade DCIS (+/− invasive disease) in the underlying lactiferous duct system. This variant of DCIS is frequently HER2 positive.

## 6. DCIS with Microinvasion

When malignant epithelial cells have breached the basement membrane and have invaded the adjacent stroma to a depth of 1 mm or less, microinvasion (MI) is said to be present. This can take the form of single cells or groups of cells and can occur singly in an area of DCIS or can occur at different points along the affected duct system [22]. When MI is multifocal the size of the individual foci should not be added together and the lesion is still staged as T1mic [23]. While it may be identified in association with all grades of DCIS it is most commonly seen with high-grade lesions. Definitive diagnosis may be problematic in cases of high-grade DCIS with extensive cancerization of lobules or cases with a prominent stromal lymphocytic infiltrate or marked stromal distortion. These difficulties can usually be resolved by the employment of a combination of additional levels, cytokeratin stains to highlight the epithelial cells and myoepithelial markers to demonstrate the presence of malignant cells beyond the boundaries of the duct space. Microinvasion particularly in association with high-grade DCIS will often prompt a sentinel lymph node biopsy and in ~10% (and in some series up to 20%) of cases lymph node metastases have been indentified, predominantly micrometastases or isolated tumor cells [24]. 

## 7. Prognostic Factors

DCIS is a recognised precursor (albeit nonobligate one) of invasive carcinoma and if left untreated small retrospective studies have shown that approximately 30% of DCIS lesions will progress to invasive cancer over a 30-year time period [25]. The rate of progression of high-grade DCIS is likely higher [26].

DCIS is treated with curative intent with a combination of surgery, +/− radiotherapy, +/− antihormonal treatment as elaborated on later, using this intensive treatment approach local recurrence (LR) occurs in 10–15% of optimally treated cases, 50% of which recur as invasive disease [27–29]. A number of clinico-pathological factors have been demonstrated to influence the rate of local recurrence (LR) following current treatment modalities. Poor prognostic factors include the following.

- *Young Age at Diagnosis.* Observational studies and randomized controlled trials have reported an increased risk of recurrent cancer in younger women. A meta-analysis concluded than women <40 years at the time of diagnosis have an 89% increase in risk of ipsilateral breast tumor recurrence (IBTR) compared to women >40 years at diagnosis [30].
- *High Tumor Grade*. Women with high versus low-grade of tumor have an increase in the odds of IBTR.
- Comedo-type Necrosis. Comedo-type necrosis has been repeatedly demonstrated to be consistently and strongly associated with increased risk of IBTR [30].
- *Large Tumour Size*. Tumor size is positively associated with higher rates of IBTR.
- *Positive Surgical Margins*. Positive surgical margins are strongly associated with the risk of IBTR. While there is no uniform definition of what an acceptable negative margin is, most people agree that a margin of 10 mm is clearly negative and a margin <1 mm is unacceptable. A margin of 10 mm or more is associated with a 98% reduction in risk of IBTR.
- *ER Negativity*. While many of the studies are small and their conclusions are often not statistically significant, a positive ER status is associated with reduced likelihood of local recurrence. A positive PR status is also associated with a tendency to lower IBTR.
- *HER2 Positivity*. HER2 positive DCIS is associated with a higher risk of recurrence. Again the studies are small.

The final pathology report must include at a minimum the following features; tumor size, distance to margins, nuclear grade, and presence and type of necrosis to allow informed decision making for patients, surgeons, and radiation oncologists. All of these factors together with patient age have been incorporated into the University of Southern California/Van Nuys Prognostic Index (USC/VNPI). This scoring system gives a score ranging from 1 to 3 for each of the four clinicopathological tumor parameters as outlined in Table 1. The authors claim that those patients with a tumor exhibiting a low USC/VNPI score (defined as a score of 4, 5 or 6) can be treated with surgery alone (LR rate of 5.4% at 12 years), whereas tumors with a higher score (7 or greater) experience a significant LR rate (>20% at 12 years) with surgery alone and hence adjuvant radiotherapy or mastectomy is required. There are many caveats to this study, not least of which are patients were not randomized to the different treatment arms (surgery alone versus surgery + radiation) and the entire excision specimen was embedded for microscopic examination. Modifications to the USC/VNPI continue to refine the outcomes for each score [9, 31–33]. 

While the reporting of ER status is variable in clinical practice it is recommended by the National Comprehensive Cancer Network (NCCN) that this will likely be a mandatory additional requirement in the near future [34]. The evaluation of ER in addition to providing prognostic information (i.e., ER positive DCIS is less likely to recur post treatment than ER negative disease) has been demonstrated to have predictive utility. In the NSABP B-24 trial patients with DCIS were randomly assigned to five years of tamoxifen (10 mg twice daily) or placebo following standard treatment with surgery and local radiotherapy. Those patients in the tamoxifen treated arm of the trial developed fewer breast cancers than those not treated with tamoxifen, 8.4% for treated versus 13.4% for untreated. Subsequent analysis of the ER status of the DCIS demonstrated that the reduction in subsequent cancer development was restricted to those patients with ER+ DCIS [35]. In this trial using a cut-off value similar to that employed for invasive breast cancer (>1% tumor nuclei staining positive) approximately 76% of DCIS samples analysed were ER positive. PR expression was additionally examined but overall PR expression was not more predictive than when ER status was considered alone [35].

## 8. Management

The standard management options for the treatment of DCIS currently include

1. lumpectomy without lymph node surgery with whole breast irradiation;
2. total mastectomy with sentinel lymph node biopsy +/− reconstruction.
3. lumpectomy without lymph node surgery or radiation.

Tamoxifen for 5 years may be considered in the adjuvant setting for those patients treated with option 1 (especially for those with proven ER positive DCIS) and option 3 [34]. The appropriate option for any given patient will depend on a variety of clinical-pathologic factors such as the age of the patient and extent of disease. Option three, the most conservative of all options, is generally only considered for patients deemed to be at very low risk of LR (<5% at 10 years) or for those patients with significant comorbid factors that may mitigate against the use of radiation therapy.

## 9. Differential Diagnosis

The differential diagnosis for DCIS varies with the grade and extent of the disease encountered.

- *Atypical Ductal Hyperplasia (ADH) and Low-Grade DCIS*. At the low-grade, minimal extent end of the spectrum the differential diagnosis lies between atypical ductal hyperplasia (ADH) and a low-grade DCIS. When a proliferation of low-grade malignant cells occupies less that two duct spaces or <2 mm in maximum extent a diagnosis of ADH should be given. This extent/size criterion does not apply to high-grade lesions.
- *Solid Low-Grade DCIS and Lobular Carcinoma In Situ (LCIS)*. Low-grade DCIS with a solid growth pattern may be difficult to be distinguished from classic LCIS on the basis of morphology alone. In these scenarios E-cadherin immunohistochemistry can usually reliably distinguish low-grade DCIS (E-cadherin positive in a circumferential membranous pattern) for LCIS (E-cadherin negative).
- *High-Grade DCIS and Pleomorphic LCIS (PLCIS)*. At the high-grade end of the spectrum distinguishing DCIS from PLCIS may be problematic. Both lesions are characterized by a proliferation of malignant pleomorphic cells with large nuclei and both often show areas of comedo-type necrosis. Morphological cues that you may be dealing with a case of PLCIS include the discohesive nature of the cells in PLCIS, the presence of intracytoplasmic vacuoles, and the predominant lobular-centric nature of the disease. In addition, classic LCIS is often seen in the vicinity of PLCIS. Once again E-cadherin IHC clearly distinguishes between high-grade DCIS (E-cadherin positive) and PLCIS (E-cadherin negative).
- *DCIS with MI *(discussed above).

## 10. Cytokeratins and DCIS

Immunohistochemical staining for cytokeratin(CK)5/6 and ER are used to great success in distinguishing a case of usual duct hyperplasia (UDH) from ADH; however, this combination is not useful in making a diagnosis of DCIS or in distinguishing low-grade DCIS from ADH [36, 37]. 

While UDH shows a mosaic-type staining pattern for CK5/6 and variable ER staining both ADH and low-grade DCIS are uniformly CK5/6 negative and ER positive and hence these two entities (ADH and low-grade DCIS) can only be reliably distinguished on the criteria of size/extent as discussed above.

A small percentage of high-grade DCIS exhibits CK5/6 positivity and ER negativity (so-called basal-like DCIS) and this should not cause confusion with UDH. 

## 11. “Intrinsic” Molecular Subtyping of DCIS

Gene expression profiling of invasive breast cancers has repeatedly shown the presence of at least 4 distinct “intrinsic” molecular subtypes of breast cancer; Luminal A, Luminal B, HER2 enriched, and basal-like [38–40]; these subtypes can also be identified at the *in situ* stage albeit at slightly different frequencies [13, 41, 42]. Using a surrogate panel of 5 immunohistochemical antibodies (ER, PR, HER2, CK5, and EGFR) the molecular subtypes can be approximated in formalin-fixed paraffin-embedded tissue sections and using these techniques a number of authors have found that HER2 enriched DCIS (~15–20% of DCIS cases) appears to be more frequent and basal-like DCIS (~4–8% of cases) are less frequent than their invasive counterparts [43–45]. The clinical significance of these findings and whether the different molecular subtypes of DCIS have different propensities to LR or progression to invasive disease is not known. 

## 12. Molecular Genetics

Many studies have been conducted to investigate the genetic alterations that underpin the development of DCIS and also the progression of DCIS to invasive carcinoma (reviewed in [46]). These studies have modified the original model of breast cancer development proposed by Wellings and Jensen. In the Wellings and Jensen model breast cancer was believed to develop over long periods of time from a normal terminal duct lobular unit (TDLU) through UDH to ADH to low-grade DCIS then high-grade DCIS and ultimately invasive cancer by the successive accumulation of random genetic alterations [47, 48]. The current model while far from being complete or universally accepted proposes two models; with low-grade DCIS being the precursor of low-grade invasive carcinoma and high-grade DCIS being the precursor of high-grade invasive disease [46, 49, 50]. To support this view low-grade *in situ* and invasive lesions (and many of their presumed precursor lesions FEA, ADH, ALH, and LCIS) are predominantly ER positive, HER2 negative lesions, have diploid or near diploid karyotypes, and are characterized oftentimes by the shared deletion of the long arm of chromosome 16 (16q) and 1q gain. In contrast, high-grade *in situ* and invasive lesions are more frequently ER negative, may be HER2 amplified, commonly are aneuploid, infrequently harbour 16q deletions but rather have recurrent and more frequent alterations (regions of gain and loss) and areas of amplifications as determined by array comparative genome hybridization (aCGH) and FISH studies. The evolution of intermediate grade disease is less clear. Currently no specific genetic alteration can predict with certainty the progression from DCIS to invasion.

## 13. Conclusions

In conclusion, DCIS is a highly heterogeneous disease that, like its invasive counterpart, is likely composed of many biologically disparate disease entities. The challenge in the future will be to distinguish those forms of DCIS likely to recur and/or progress to invasive disease from the more indolent forms of the disease and to tailor imaging and treatment decisions accordingly. 

## Figures and Tables

**Table 1:** Scoring system for the University of Southern California/Van Nuys Prognostic Index.

Score	1	2	3
Size	≤15 mm	16–40 mm	>40 mm
Margin	≥10 mm	1–9 mm	<1 mm
Class	Grade 1/2 no necrosis	Grade 1/2 with necrosis	Grade 3
Age	>60	40–60	<40
